# Mapping codon usage of the translation initiation region in porcine reproductive and respiratory syndrome virus genome

**DOI:** 10.1186/1743-422X-8-476

**Published:** 2011-10-21

**Authors:** Jun-hong Su, Xiao-xia Ma, Ya-li He, Ji-dong Li, Xu-sheng Ma, Yong-xi Dou, Xue-nong Luo, Xue-peng Cai

**Affiliations:** 1 State Key Laboratory of Veterinary Etiological Biology, Lanzhou Veterinary Research Institute, Chinese Academy of Agricultural Sciences, Lanzhou, 730046, PR China; 2 College of Life Science and Engineering, Northwest University for Nationalities, Lanzhou, 730030, PR China; 3 The School of Public Health, Lanzhou University, Lanzhou, 730000, RP China; 4 School of Agriculture, Ningxia University, Yinchuan, 750021, RP China

**Keywords:** PRRSV, codon usage bias, translation initiation region, translation efficiency, translation selection

## Abstract

**Background:**

Porcine reproductive and respitatory syndrome virus (PRRSV) is a recently emerged pathogen and severely affects swine populations worldwide. The replication of PRRSV is tightly controlled by viral gene expression and the codon usage of translation initiation region within each gene could potentially regulate the translation rate. Therefore, a better understanding of the codon usage pattern of the initiation translation region would shed light on the regulation of PRRSV gene expression.

**Results:**

In this study, the codon usage in the translation initiation region and in the whole coding sequence was compared in PRRSV ORF1a and ORFs2-7. To investigate the potential role of codon usage in affecting the translation initiation rate, we established a codon usage model for PRRSV translation initiation region. We observed that some non-preferential codons are preferentially used in the translation initiation region in particular ORFs. Although some positions vary with codons, they intend to use codons with negative CUB. Furthermore, our model of codon usage showed that the conserved pattern of CUB is not directly consensus with the conserved sequence, but shaped under the translation selection.

**Conclusions:**

The non-variation pattern with negative CUB in the PRRSV translation initiation region scanned by ribosomes is considered the rate-limiting step in the translation process.

## Introduction

Porcine reproductive and respiratory syndrome virus (PRRSV) infection causes serious disease in swine populations with a series of clinical consequences, such as high mortality, reproductive failure, post-weaning pneumonia and growth reduction [1,2]. Based on its serological characteristics, PRRSV has two main serotypes, which named the Northern American isolate (US) and the European isolate (EU), respectively [3-7]. PRRSV is an enveloped, single-stranded positive-sense RNA virus with a genome size of about 15.4kb and classified into the order *Nidovirales *of family *Arteriviridae *[8,9]. The PRRSV genome contains ORF1a, encoding papain-like cysteine protease, ORF1b, encoding RNA dependent RNA polymerase, ORF2-6, encoding envelop proteins, and ORF7, encoding the nucleocapsid protein [10-13]. Despite a well-organization of the ORFs within the single RNA genome, viral proteins are in fact encoded from subgenomic RNAs that are likely generated through a discontinuous transcription mechanism [12,14]. Therefore, each subgenomic RNA could be translated at different translation rates that are regulated by codon usage bias (CUB). Because the faster a polypeptide chain is completed, the more rapid the ribosomes return to initiate and complete another polypeptide chain. The relationship between the efficiency of translation initiation and the level of gene expression has been well-established in many species [15-19]. Moreover, when the distance between the initiation codon and the non-preferential site is less than 50-60 positions (codons), the ribosomes can be blocked at the non-preferential positions to shape a queue of ribosomes [20].

It is generally considered that the alternative synonymous codons are not used with equal frequencies among organisms, and the codon usage pattern plays a role in genes expressed at higher levels [21-30]. Jacques and Dreyfus proposed that the translation initiation site is a rate-limiting factor for gene expression [31]. Nevertheless, a regulatory relationship, which is thought to be mediated by preferential codons, between CUB and translation efficiency for individual genes is challengeable [32,33]. This suggested that a heterogonous gene is not necessarily expressed at a low level simply because its codons are infrequently translated by the host cell. There is a codon bias with respect to intragenic codon bias in the initial sequences of genes for which major proteins are strikingly different from their downstream codon bias. It is found that the translational initiation region plays an important role in regulating the translational efficiency and the pattern of synonymous codon usage varies in different regions along a coding sequence [34,35]. This indicated that the alternative synonymous codon usage might be related with gene function, protein structure and translation efficiency. In this study, we focus on the pattern of CUB in the translation initiation region of PRRSV as well as the characteristics of the synonymous codon usage at each position in the target region, since the interest in the pattern of CUB has been aroused by its potential relevance to the translational efficiency of PRRSV subgenomic RNAs. And the frequency of non-preferential codons usage in the target region is investigated in order to evaluate the role of translation selection on the formation of negative CUB pattern.

## 2. Materials and methods

### 2.1. Sequences data and the synonymous codon usage value

The 13 complete RNA sequences of PRRSV were downloaded from the National Center for Biotechnology Information (NCBI) http://www.ncbi.nlm.nih.gov/Genbank/ and the synonymous codon usage values (SCUV) for this virus were reported previously [30]. Multiple alignment analyses were performed with the Clustal W (1.7) method of DNAStar software (7.0) for windows. The translation initiation regions (the 1^st ^to the 50^th ^residue) of ORF1a, ORF2, ORF3, ORF4, ORF5, ORF6 and ORF7 were used as targets for alignment analysis respectively.

### 2.2. The calculation of codon usage bias

To calculate CUB, it is supposed that statistically equal and random usage of all available synonymous codons was the "neutral point" (RSCU_0 _= 1.00) for the development of serotype-specific codon usage [19]. CUB:

$$CUB=\overset{n}{\underset{i=1}{\sum }}\left(RSCU_{ij}-RSCU_{0}\right)∕n$$

More simply, *CUB *is the average value of difference between *RSCU_ij _*and *RSCU_0 _*at each position of the target region. *n *represents all codons appearing in this position. When all *RSCU *values according to a particular position in the target region are *RSCU_0_, CUB *is equal to zero. It means that there are few preferential or non-preferential codons existing at this position. In contrast, when *CUB *value is much more deviation than *RSCU_0_*, codons with CUB are preferentially chosen at a particular position.

### 2.3. Analysis of codon usage characteristic of the translation initiation region

We analyzed the codon usage characteristics of the translation initiation region depending on *R *values, where the *R *value, computed as the ration ***R *= (n*_i_*/N*_i_*)/(n/N)**, represents the relative abundance for a particular codon in the translation initiation region. n_i _represents the total number of a particular codon within the 1^st ^to *i*^th ^amino acids, N*_i _*represents the total number of corresponding amino acid in the 1^st ^to *i*^th ^amino acid ones, n is the total number of a certain codon within the whole coding sequence, and N is the total number of corresponding amino acids within the whole coding sequence. When *R *value is equal to 1.00, it means that the frequency of this codon in the target region is equal to the frequency of this codon in the whole coding sequence; when *R *value is lower than zero, it implies that the frequency of this codon in the target region is lower than that of the whole coding sequence; when *R *value is higher than zero, it suggests that the frequency of this codon is higher than that of the whole coding sequence.

### 2.4. Aanalysis of characteristics of positions with negative CUB in the target regions

To substantiate the characteristics of codon usage for positions with negative CUB in the target regions, we analyzed the target positions depending on the data, (i) the variations of codons and amino acids, (ii) *R *values for codons of the target positions.

## 3. Results

### 3.1. Multiple alignment analysis

The consensus amino acid sequence is based on the comparison of the strains in previous study [30]. The positions of amino acid conservation are listed in Table 1. The conservation of amino acid usage in translation region was analyzed. For ORF1a, 94% of amino acids in the target region of US serotype were invariant; 70% in the target region of EU serotype were conserved. For ORF2, 78% of amino acids were invariant in US serotype; 60% were invariant in EU serotype. Non-conserved amino acids scattered into the target regions of both US and EU serotypes. For ORF3, 74% of amino acids were invariant in US serotype; 60% were invariant in EU serotype, the most conserved amino acids tended to exist in the C' termination of the target regions of both US and EU serotypes. For ORF4, 76% of amino acids were invariant in US serotype; 72% were invariant in EU serotype. Non-conserved amino acids scattered in the flank of the target regions of both US and EU serotypes. For ORF5, 72% of amino acids were invariant in US serotype; 66% were invariant in EU serotype. Non-conserved amino acids scattered into the target regions of both US and EU serotypes. For ORF6, 96% of amino acids were invariant in US serotype; 82% were invariant in EU serotype, and non-conserved amino acids had a tendency to exist in the N' termination. For ORF7, 90% of amino acids were invariant in US serotype; 76% were invariant in EU serotype, and conserved amino acids scattered into the target region compared with that of US serotype. The various extents of the conserved amino acids encoded by ORFs of PRRSV suggested that these residues played an important role in virus biology.

**Table 1:** The positions of invariant amino acids in the translation initiation region

ORF	Serotype	The position of amino acid conservation in the translation initiation region
ORF1a	US	The 2^nd ^to 17^th^, 19^th ^to 34^th^, 36^th ^to 41^st^, 43^rd ^to 50^th^
	
	EU	The 3^rd^, 6^th ^to 13^th^, 15^th ^to 18^th^, 20^th ^to 23^rd^, 25^th ^to 28^th^, 30^th ^to 32^nd^, 34^th^, 35^th^, 39^th ^to 41^st^, 44^th^, 46^th^, 48^th ^to 50^th^

ORF2	US	The 2^nd ^to 4^th^, 6^th^, 8^th^, 11^th ^to 13^th^, 15^th ^to 22^nd^, 25^th ^to 31^st^, 33^rd ^to 41^st^, 43^rd ^to 44^th^, 46^th ^to 49^th^
	
	EU	The 2^nd ^to 4^th^, 7^th^, 12^th ^to 13^th^, 15^th^, 18^th^, 20^th^, 22^nd^, 24^th ^to 27^th^, 32^nd ^to 37^th^, 40^th^, 41^st^, 43^rd ^to 49^th^

ORF3	US	The 4^th^, 5^th^, 7^th^, 9^th ^to 12^th^, 14^th^, 16^th ^to 19^th^, 21^st^, 22^nd^, 24^th ^to 26^th^, 29^th^, 31^st^, 33^rd ^to 47^th^, 49^th^, 50^th^
	
	EU	The 2^nd^, 4^th^, 15^th^, 18^th^, 20^th^, 24^th ^to 26^th^, 28^th^, 31^st ^to 50^th^

ORF4	US	The 2^nd^, 6^th ^to 8^th^, 10^th ^to 12^th^, 14^th^, 17^th ^to 31^st^, 33^rd^, 34^th^, 36^th ^to 41^st^, 44^th^, 46^th ^to 50^th^
	
	EU	The 3^rd^, 4^th^, 6^th^, 7^th^, 9^th^, 12^th^, 13^th^, 17^th ^to 32^nd^, 34^th^, 36^th ^to 39^th^, 41^st^, 42^nd^, 44^th^, 46^th ^to 48^th^, 50^th^

ORF5	US	The 2^nd^, 6^th ^to 8^th^, 10^th^, 12^th^, 14^th^, 15^th^, 18^th ^to 23^th^, 26^th ^to 28^th^, 30^th ^to 34^th^, 36^th^, 39^th ^to 46^th^, 48^th ^to 50^th^
	
	EU	The 3^rd^, 4^th^, 6^th^, 7^th^, 14^th ^to 16^th^, 18^th^, 19^th^, 21^st^, 24^th^, 26^th ^to 28^th^, 30^th ^to 34^th^, 36^th^, 39^th ^to 46^th^, 48^th ^to 50^th^

ORF6	US	The 2^nd ^to 9^th^, 11^th ^to 15^th^, 17^th ^to 50^th^
	
	EU	The 2^nd^, 4^th^, 5^th^, 7^th^, 8^th^, 15^th ^to 22^nd^, 24^th ^to 50^th^

ORF7	US	The 2^nd ^to 10^th^, 12^th ^to 14^th^, 16^th ^to 45^th^, 47^th^, 50^th^
	
	EU	The 2^nd^, 3^rd^, 5^th^, 6^th^, 8^th ^to 10^th^, 12^th^, 15^th ^to 21^st^, 23^rd ^to 28^th^, 30^th^, 31^st^, 33^rd^, 35^th ^to 38^th^, 42^nd ^to 50^th^

### 3.2. Characteristics of codon usage bias in the target regions

**Figure 1:**
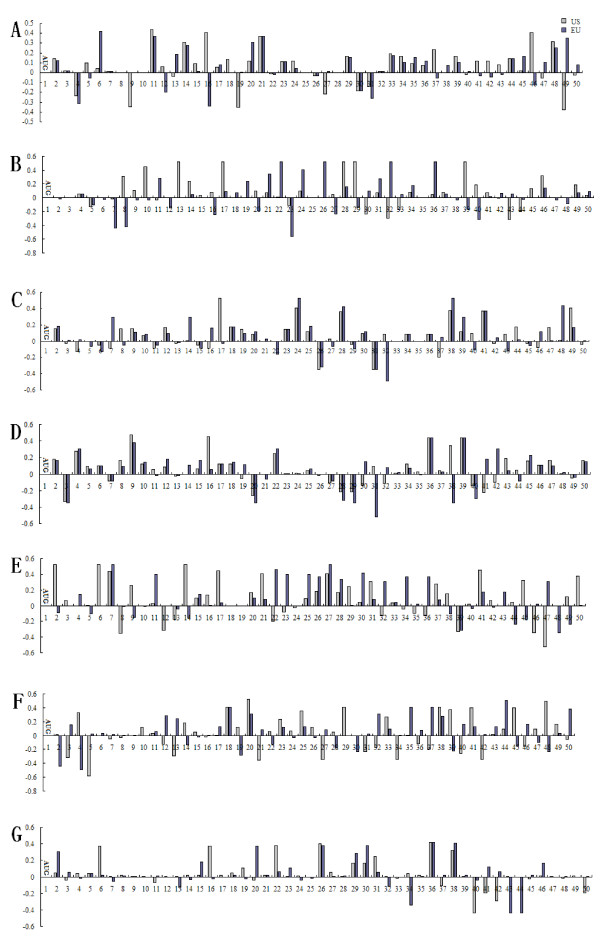
**The CUB degree of translation initiation region in PRRSV ORFs, the white bar represents US serotype while the gray represents EU**. **A**, ORF1a; **B**, ORF2; **C**, ORF3; **D**, ORF4; **E**, ORF5; **F**, ORF6; **G**, ORF7.

The bars of all positions in the translation initiation region represented the CUB degree (Figure 1). Although different invariant degrees of the amino acids exist in the target regions between US and EU serotypes, the similar patterns of codon usage are present in the target regions of both US and EU serotypes (Table 2). For ORF1a, 58% of positions possess the similar pattern of codon usage in the target regions of both serotypes. Although the two target regions corresponding to both the US and EU serotypes have a significant difference to the conservation in obvious amino acids, a large size of the similar patterns of codon usage exist in the target region and the most positions possessed the positive codon usage bais (Figure 1A). For ORF2, 34% of positions have the similar pattern of codon usage, and the positions in the N-terminal fragment had a tendency to choose low codon bias. It was also observed that the number of the positions with the negative codon usage bias for US serotype was more than that of EU serotype (Figure 1B). For ORF3, 62% of positions have the similar pattern of codon usage (Figure 1C). For ORF4, 72% positions contain the similar pattern of codon usage (Figure 1D). For ORF5, 40% of positions have the similar pattern of codon usage, and these positions with the similar pattern of codon usage do not appear to exist near the N' termination (Figure 1E). For ORF6, 26% of positions which contain the similar pattern of codon usage do not exist near the N' termination (Figure 1F). For ORF7, 44% of positions have the similar pattern of codon usage, and the most positions with low codon usage bias tend to exist near the N-terminal fragment (Figure 1G).

**Table 2:** The similar pattern of codon usage in the target regions in both US and EU serotypes

ORFs	The positions corresponding to similar codon usage pattern in the target region
ORF1a	the 2^nd ^to 4^th^, 6^th ^to 8^th^, 10^th^, 11^th^, 14^th^, 15^th^, 17^th^, 20^th ^to 24^th^, 26^th^, 29^th ^to 36^th^, 39^th^, 44^th^, 45^th^, 48^th^
ORF2	the 3^rd ^to 5^th^, 14^th^, 17^th^, 21^st^, 23^rd^, 24^th^, 28^th^, 31^st^, 34^th^, 36^th^, 37^th^, 41^st^, 44^th^, 46^th^, 49^th^, 50^th^
ORF3	the 2^nd^, 6^th^, 9^th ^to 15^th^, 18^th ^to 20^th^, 23^rd ^to 26^th^, 28^th ^to 31^st^, 33^rd ^to 36^th^, 38^th^, 39^th^, 41^st^, 44^th^, 45^th^, 47^th ^to 49^th^
ORF4	the 2^nd ^to 10^th^, 12^th^, 13^th^, 15^th ^to 18^th^, 20^th^, 22^nd ^to 25^th^, 27^th ^to 29^th^, 33^rd^, 34^th^, 36^th^, 37^th^, 39^th^, 40^th^, 43^rd^, 45^th^, 46^th^, 47^th ^to 50^th^
ORF5	the 7^th^, 11^th^, 13^th^, 15^th ^to 17^th^, 20^th^, 21^st^, 25^th ^to 31^st^, 33^rd^, 37^th^, 39^th^, 41^st^, 50^th^
ORF6	the 11^th^, 17^th^, 18^th^, 20^th^, 23^rd^, 25^th^, 33^rd^, 38^th^, 41^st^, 43^rd^, 44^th^, 49^th^
ORF7	the 2^nd^, 5^th^, 6^th^, 8^th^, 9^th^, 15^th^, 18^th^, 21^st ^to 23^rd^, 26^th ^to 31^st^, 36^th^, 38^th ^to 40^th^, 46^th^

The various extents of the conserved pattern of codon usage for their positions in PRRSV ORFs suggest that CUB associated with these positions might modulate the corresponding gene expression.

### 3.3. The rate of codon usage frequency in the translation initiation region to that of the whole coding sequence

The *R *value for each codon was calculated and listed in Table 3. A higher *R *value indicated more preferential usage in the translation initiation site than that of the whole coding sequence. *CUB_ij _*value for each codon was listed in Table 4. Depending on the data from Table 3, 4 and comparison with the whole coding sequence of PRRSV, for ORF1a, the codons with negative CUB, namely GCA (Ala), GCG (Ala), CAA (Gln), AGU (Ser), ACA (Thr) and ACG (Thr), were more preferentially chosen in the target region for both serotypes; for ORF2, the codons, namely UGU (Cys), AUA (Ile), AAA (Lys), CCG (Pro), AGU (Ser) and UCG (Ser), were more preferentially used; for ORF3, the codons, namely UGU (Cys), AGC (Ser) and ACG (Thr), were more preferentially chosen; for ORF4, the codons, namely GAC (Asp), UUC (Phe), AGU (Ser) and UCG (Ser), were more preferentially chosen; for ORF5, the codons, namely UGU (Cys), CCG (Pro), UCG (Ser) and ACG (Thr), were more preferentially chosen; for ORF6, the codons, namely CAA (Gln), AUA (Ile) and CUA (Leu), were more preferentially used; for ORF7, the codons, namely GGA (Gly) and AAA (Lys), were more preferentially chosen. Due to these non-preferential codons, ribosomes might be stalled by them to regulate the efficiency of gene translation.

**Table 3:** Preferentially used codons in the target region in US and EU serotypes of PRRSV

	ORF1a		ORF2			ORF3		
	
Codon	US	EU	US		EU	US	EU	
^a^GCA	^b^1.69	^b^1.43	0		0.91	0.15	0.64	
GCC	1.00	0.98	1.42		2.16	0	0.91	
^a^GCG	^b^2.25	^b^1.34	0		0	^b^2.17	0	
GCU	0	0.76	1.03		0.83	2.94	1.88	
^a^AGA	0	0	0.5		^b^1.37	0	0	
AGG	1.22	0	0		0	3.72	0	
^a^CGA	^b^2.42	0	0		0	0	0	
CGC	0	0	0		0	0	2.05	
CGG	3.07	5.63	3.79		1.14	0	0	
^a^CGU	0	0	0		^b^1.07	0.47	^b^2.90	
AAC	0.43	0.90	1.86		0	0.12	1.01	
^a^AAU	^b^1.72	0.68	0.40		0	^b^1.35	1.00	
^a^GAC	0	^b^1.56	0		0	0	0	
GAU	2.31	0.37	0		1.26	0.38	0.25	
UGC	1.08	1.62	1.25		0	0.36	0.18	
^a^UGU	0.92	0.50	^b^1.01		^b^1.51	^b^1.44	^b^1.43	
^a^CAA	^b^1.89	^b^1.08	0.50		0.86	0	0	
CAG	0.17	0.92	0		1.21	0	2.15	
^a^GAA	0.55	0.28	0		0	^b^1.50	0	
GAG	1.30	1.38	0		1.14	0.27	1.30	
^a^GGA	0	0.39	0		^b^2.46	0	0	
GGC	1.40	1.63	3.13		0	1.51	3.82	
GGG	2.00	1.16	0		0.29	0	0	
GGU	0.15	0.48	1.40		1.25	2.58	0.42	
^a^CAC	0	0	0		^b^1.58	0	0.72	
CAU	0	0	0		0.21	1.41	1.32	
^a^AUA	^b^8.26	0	^b^2.55		^b^3.19	0	0	
AUC	0	0	0		0	0	2.58	
AUU	0	0.59	0		0	4.31	0	
^a^CUA	^b^2.32	0.24	0.32		0	0	^b^4.42	
CUC	1.90	1.87	0		0	1.84	0.77	
CUG	0.92	0	0		0.65	1.39	0.43	
CUU	1.01	1.34	0.66		0.24	0	1.02	
^a^UUA	0.28	0.22	0.64		^b^3.54	0	0	
UUG	0	0.80	1.99		2.04	0.74	1.29	
^a^AAA	0	0	^b^1.27		^b^2.64	0	0	
AAG	0	0	0		0	0	0	
^a^UUC	^b^1.01	0.48	0.17		^b^1.09	0.99	0.95	
UUU	1.01	1.46	1.14		0.78	1.02	1.07	
CCA	0	0.98	1.50		1.04	0	4.51	
CCC	2.27	0.11	0		0	0.39	0	
^a^CCG	0	^b^2.43	^b^1.36		^b^1.07	^b^3.79	0	
CCU	1.01	0.81	0		3.21	0	0.24	
^a^AGC	0.42	^b^1.56	0		0.43	^b^2.02	^b^1.26	
^a^AGU	^b^6.36	^b^8.28	^b^4.94		^b^4.13	0	^b^1.87	
UCA	1.89	1.68	0.28		0.41	0	1.17	
UCC	0.21	0.98	0.09		0.60	0.83	0.59	
^a^UCG	0	0	^b^1.58		^b^1.70	0	^b^1.30	
UCU	2.79	1.33	1.16		0.63	2.21	1.22	
^a^ACA	^b^1.97	^b^1.48	^b^6.17		0	^b^1.24	0.70	
ACC	0.89	0.58	0		0	0	0.70	
^a^ACG	^b^2.22	^b^1.49	0		^b^1.83	^b^1.74	^b^2.11	
^a^ACU	0	^b^1.06	0		0	0.96	0.82	
UAC	1.33	0	0		1.36	1.32	2.03	
^a^UAU	0.37	0.30	^b^1.77		0.53	0	0.11	
^a^GUA	0	^b^2.67	0		^b^4.79	0	0	
GUC	0.77	1.77	3.78		0.33	0.11	0.33	
GUG	1.71	0.31	0		2.04	1.88	0	
GUU	0.76	0.53	2.96		0	1.08	4.14	
	
	ORF4		ORF5		ORF6		ORF7	
	
Codon	US	EU	US	EU	US	EU	US	EU

^a^GCA	^b^1.25	0.16	0	0	0	^b^1.60	0	^b^1.09
GCC	0.55	0.96	1.91	3.08	0.65	1.69	1.22	1.75
^a^GCG	^b^1.27	^b^1.09	0.85	0.57	^b^1.60	0	0	0
GCU	0.98	1.20	1.35	0	2.24	0.21	1.50	0.74
^a^AGA	0	0	0	^b^3.69	0	0	^b^2.78	0.71
AGG	0	0	0	0.15	0	0	0.17	2.19
^a^CGA	0	0	^b^7.83	0	^b^4.58	0.99	0	0.33
CGC	0	0	0	0	2.29	5.79	0	0.75
CGG	0	0	0	0	0	0.23	0	0.29
^a^CGU	0	0	0	^b^1.80	0	0	0	0
AAC	3.67	1.36	1.34	1.42	0	0.31	1.09	1.42
^a^AAU	0	0.56	0	0.20	0.29	0.86	0.89	0.83
^a^GAC	^b^1.38	^b^1.39	0.58	0.18	^b^1.33	0.64	0.50	0
GAU	0.50	0.69	0	2.65	0.67	0.86	0.75	0
UGC	0.64	0.67	0.82	0.99	0.44	1.38	1.92	1.00
^a^UGU	^b^1.41	0.99	^b^1.13	^b^1.02	^b^1.33	0.24	0	0.29
^a^CAA	0.42	^b^1.14	0.25	1.00	^b^1.50	^b^1.38	0.64	0.78
CAG	1.46	0.95	2	0	0.33	0	1.13	1.07
^a^GAA	0.50	0	0	0	0.17	0.45	0	0
GAG	0.19	2.14	1.49	0.29	0	0	0	0
^a^GGA	0	0	0	0	0	0.84	^b^1.31	^b^1.14
GGC	0.63	0	1.63	1.98	0.79	0.77	1.05	0.89
GGG	0	0	1.03	0.54	1.87	1.97	0.63	0.85
GGU	4.00	3.33	0.17	0	0	0.56	1.35	1.11
^a^CAC	0	0	0	^b^2.90	0.78	^b^2.05	0	0
CAU	0	1.48	1.94	0	1.46	0	0	0
^a^AUA	0	0	0	^b^2.58	^b^1.12	^b^1.86	0	^b^3.29
AUC	1.26	1.24	1.07	0.12	1.19	0.90	1.46	0
AUU	0.73	1.20	1.34	0.37	0.80	0.08	0	0
^a^CUA	0	^b^2.26	^b^1.37	0	^b^1.66	^b^1.35	0	0
CUC	1.52	1.15	0.60	0	0.19	0.74	0	0
CUG	0	1.14	0.70	0.99	1.19	1.48	1.46	1.78
CUU	1.19	1.81	0.62	0.77	1.27	0.94	0	0
^a^UUA	0	0.23	0.55	0	0.77	0.16	0	0
UUG	1.50	0.19	1.69	2.16	0.73	0.84	0.22	1.05
^a^AAA	^b^1.21	0.20	^b^1.34	1.00	0	0.31	^b^1.08	^b^1.21
AAG	0.69	1.82	0.23	0.38	2.43	1.32	0.98	0.86
^a^UUC	^b^1.28	^b^1.18	^b^1.88	0.99	1.00	0.42	0	0
UUU	0.76	0.12	0.84	0.98	0.87	1.49	0	0
CCA	3.83	0	0	0.76	3.00	0	2.25	1.85
CCC	0.40	1.67	0	0	0	0.79	0	0.59
^a^CCG	0	0.43	^b^4.25	^b^1.29	0	0	^b^2.00	0.33
CCU	0	0.24	0.17	0	0	1.65	0	0.50
^a^AGC	^b^1.34	0	^b^1.69	0.57	0.98	^b^3.10	1.00	^b^2.47
^a^AGU	^b^3.67	^b^2.57	0	0	^b^4.28	0	0	^b^2.51
UCA	4.31	0.64	0	0	2.20	0	0	2.99
UCC	0.78	0	0.32	1.17	0.79	0	1.35	1.26
^a^UCG	^b^1.91	^b^1.95	^b^1.58	^b^1.48	^b^2.20	0	0	0
UCU	0.47	0.86	1.45	1.78	2.02	0.31	0	0
^a^ACA	0	0	0	^b^1.32	0.17	^b^1.76	0	^b^1.63
ACC	1.78	1.41	0.98	0.67	0.83	0.56	0	0
^a^ACG	0	^b^1.69	^b^6.79	^b^2.13	^b^2.35	0	0	0
^a^ACU	0.78	0.21	0	0.53	0.18	0.43	0	0
UAC	0	0	1.80	1.11	0.67	1.05	0	0
^a^UAU	0	0	0.48	0.90	^b^1.50	0.55	0	0
^a^GUA	0	0	0	0	0.98	0.27	0	0
GUC	0.92	1.04	0.34	0	0	0	1.72	2.96
GUG	0.52	0.10	2.79	0	2.10	2.29	0	0
GUU	2.25	1.77	0	0	0	0	0	0

^a ^presented the non-preferential codon.

^b ^presented that the non-preferential codon was more preferentially chosen in the translation initiation region than that of the whole coding sequence.

**Table 4:** Synonymous codon usage bias for the whole coding sequence of PRRSV

^**a**^**AA**	Codon	^**b**^**CUB**_***ij***_	**AA**^**a**^	Codon	^**b**^**CUB**_***ij***_
Ala	GCA	-0.143	Leu	CUA	-0.490
	GCC	0.309		CUC	0.164
	GCG	-0.350		CUG	0.377
	GCU	0.184		CUU	0.096
Arg	AGA	-0.107		UUA	-0.670
	AGG	0.092		UUG	0.522
	CGA	-0.258	Lys	AAA	-0.003
	CGC	0.411		AAG	0.003
	CGG	0.012	Phe	UUC	-0.083
	CGU	-0.149		UUU	0.083
Asn	AAC	0.040	Pro	CCA	0.047
	AAU	-0.04		CCC	0.059
Asp	GAC	-0.045		CCG	-0.198
	GAU	0.045		CCU	0.092
Cys	UGC	0.001	Ser	AGC	-0.128
	UGU	-0.001		AGU	-0.194
Gln	CAA	-0.018		UCA	0.075
	CAG	0.018		UCC	0.418
Glu	GAA	-0.116		UCG	-0.317
	GAG	0.116		UCU	0.145
Gly	GGA	-0.441	Thr	ACA	-0.031
	GGC	0.369		ACC	0.439
	GGG	0.016		ACG	-0.347
	GGU	0.056		ACU	-0.061
His	CAC	-0.153	Tyr	UAC	0.174
	CAU	0.153		UAU	-0.174
Ile	AUA	-0.234	Val	GUA	-0.635
	AUC	0.166		GUC	0.110
	AUU	0.068		GUG	0.406
				GUU	0.118

^a ^mean amino acid

^b^The CUB*_ij _*value was calculated following by the equation: **CUB*_ij _*= RSCU*_ij_*-RSCU_0_**, and *RSCU_ij _*value came from the previous study [30].

### 3.4. The characteristics of codon usage for the target positions

The positions with negative CUB do not always use the codons with negative CUB, and the *R *value for the codons with negative CUB vary compared with *R *= 1.00. However, some target positions contain the codons with negative CUB and *R *values > 1.00, suggesting that some new characteristics might influence the translation efficiency of the corresponding coding sequence. In translation initiation region of ORF1a, the non-preferential codons (*R *value > 1.00) are preferentially used in the 4^th ^(US and EU serotypes), 9^th ^(US), 12^th ^(EU), 19^th ^(US), the 22^nd ^(US and EU), 27^th ^(US), 31^st ^(US and EU) and 40^th ^(US), while some non-preferential codons, which have *R *value < 1.00 or R value > 1.00, exist in the 16^th ^(EU) and 30^th ^(US and EU) positions. For ORF2, the non-preferential codons are more preferentially used in the 7^th ^(EU), 8^th ^(EU), 9^th ^(EU), 11^th ^(US), 20^th ^(EU), 27^th ^(EU), 30^th ^(US), 33^rd ^(US), 40^th ^(EU), 43^rd ^(EU), 44^th ^(US) and 48th (EU) positions, while some non-preferential codons with *R *value > 1.00 or *R *value < 1.00 exist in the 12^th ^(US) position. For ORF3, non-preferential codons (*R *value > 1.00) exist in the 4^th ^(US), 13^th ^(US and EU), 17^th ^(EU), 26^th ^(US and EU), 31^st ^(US and EU), 32^nd ^(EU) and 37^th ^(US) positions, while the non-preferential codons with *R *value > 1.00 or *R *value < 1.00 are used in the 5^th ^(EU), 6^th ^(US and EU), 7^th ^(US), 11^th ^(US and EU),16^th ^(US) and 43^rd ^(EU) positions. For ORF4, the non-preferential codons with *R *value > 1.00 are used in the 3^rd ^(US and EU), 7^th ^(US and EU), 20^th ^(US and EU), 27^th ^(US and EU), 28^th ^(US and EU), 29^th ^(US and EU), 38^th ^(EU),40^th ^(US and EU), 41^st ^(US), 44^th ^(EU) and 49^th ^(US and EU), while some non-preferential nodons with *R *value > 1.00 or *R *value < 1.00 are used in the 31^st ^(EU) position. For ORF5, the non-preferential nodons with *R *value > 1.00 are used in the 9^th ^(EU), 12^th ^(US), 14^th ^(EU), 22^nd ^(US) 23^rd ^(US), 32^nd ^(US), 36^th ^(US), 39^th ^(EU), 40^th ^(EU), 44^th ^(EU), 48^th ^(EU) and 49^th ^(EU), while non-preferential codon with *R *value > 1.00 or *R *value > 1.00 are used in the 8^th ^(US), 24^th ^(US), 46^th ^(US) and 47^th ^(US) positions. For ORF6, the non-preferential codons (R value > 1.00) are used in the 3^rd ^(US), 4^th ^(EU), 7^th ^(US), 13^th ^(US), 14^th ^(EU), 15^th ^(EU), 19^th ^(EU), 21^st ^(US), 22^nd ^(EU), 24^th ^(EU), 26^th ^(EU), 27^th ^(US), 30^th ^(EU), 31^st ^(EU), 32^nd ^(US), 37^th ^(US), 40^th ^(US), 45^th ^(EU) and 48^th ^(EU) positions, while some non-preferential codon (*R *value < 1.00 or *R *value > 1.00) are used in the 2^nd ^(EU), 5^th ^(US and EU), 46^th ^(US) and 50^th ^(US) positions. For ORF7, the non-preferential codons (R value > 1.00) are chosen in the 11^th ^(US), 32^nd ^(US), 40^th ^(US and EU), 41^st ^(US), 43^rd ^(EU), 44^th ^(EU), 48^th ^(US) and 50^th ^(US) positions, while some non-preferential codon (*R *value > 1.00 or *R *value < 1.00) are used in the 3^rd ^(US), 24^th ^(EU), 25^th ^(EU) and 35^th ^(US) positions. The rest positions with negative CUB do not arise from the existence of non-preferential codons but contain some preferential codons (CUB > 0), implying that these positions do not affect the efficiency of gene translation. The degeneracy of the genetic code enables the same amino acid sequences to be encoded and translated in different ways. However, the synonymous codon usage is not purely random.

## 4. Discussion

RNA virus possesses high mutation rates and therefore virus populations exist as dynamic and complex mutant distributions [36-41]. However, the redundant intensity of mutation has deleterious effects on the viral fitness. Thus, the robustness of viral sequences can perform a reduced sensitivity to perturbations affecting phenotypic expression. The balance between the high mutations and the robustness produce a dynamic population pool, termed as 'quasispecis' [36,42]. As to comparative genomics, it is generally accepted that sequences with a crucial function are conserved among different but related organisms [43-45]. In addition, Akashi found that the frequency of preferential codons is significantly higher at the conserved amino acid positions than that at the non-conserved amino acid positions among different *Drosophila *species, suggesting that translation selection favors the conserved pattern of synonymous codon usage to enhance the accuracy of gene expression [46]. A lot of experimental data have shown that rates of chain elongation during translation of proteins are not uniform [47]. Non-uniform character of distribution of codons with different usage frequencies along mRNA is assumed to be a main factor to modulate the translation rate. Extensive studies have been carried out previously on the determination of the translation rates and the overall level of gene expression for certain individual codons [48-52]. From this research, we observed that the conserved pattern of codon usage did not simply follow the corresponding positions in the conserved sequence fragment, suggesting that the conservation of codon usage within a gene sequence have an important function in modulating its translational rate. The positions with the conserved positive CUB enhance the accuracy and efficiency of their gene translation. It has been observed that preferential codons can reduce the frequency of amino acid misincorporations, resulting in an approximately 10-fold increase of protein products over non-preferential codons for the same amino acid [53]. However, the positions with negative CUB in the translation initiation region of each PRRSV subgenomic RNA are not ignored. Because these positions are likely to regulate the translation initiation rate to generate the target product with high activity. Lithwich and Margalit reported that CUB is most highly associated with protein expression and is most conserved [26]. Once a significant number of gene sequences have been obtained, it will be taken into consideration that biased codon usage can regulate the expression levels of individual genes by modulating the rates of polypeptide elongation [21,54-58]. Komar pointed out that although preferential codons enable the corresponding gene to be translated efficiently, the non-preferential codons replaced by the corresponding preferential codons can regulate the gene expression to perform the precise protein folding [59]. Lavner and Kotlar indicated that translation selection may shape codon bias pattern, not only to increase translation efficiency by favoring preferential codons in highly expressed genes, but also to decrease translation rate by favoring non-optimal codons in lowly expressed ones [60]. A relationship between the translation efficiency and CUB have been reported that it can lead to link between the protein folding by modulating the translational rate and the synonymous codon usage bias [47,61-65]. The nucleotide sequences around the N-terminal region of the protein appear to be particularly sensitive to the presence of rare codons [66,67]. Our data showed that some positions in the translation initiation regions of ORFs tended to preferentially choose non-preferential codons which were more preferentially used in these regions than the whole coding sequences. This phenomenon suggested that the determinant of the invariant pattern of codon usage is not only correlated with the conserved sequence, but also dependent of the translation selection. As codon usage pattern comprised of preferential and non-preferential codons contributes to different translation rates, it is possible to change the local translation rates of a gene by suitable selection of its synonymous codons. A gene sequence with non-preferential codons intends to encode turns, loops and domain linkers within its protein structure through the limited step to the translation rate [47,63,64,68]. Taken together, under the translation selection, the conserved non-preferential codons in the translation initiation regions of PRRSV may affect the translation efficiency so as to maintain the normal biological functions of their target products. Komar and Jaenicke indicated that the non-preferential coodns play an important role in maintaining the normal function or activity of *CAT *product [68]. It shows the importance of non-preferential codons to the formation of the target products. As non-preferential codons or even one aggregating near the translation initiation codon can decrease translation rate arising from the limitation of availability of tRNAs depending on the host cell [69], the view that non-preferential codons probably have a negative effect on gene expression can be explained by the 'minor codon modulator hypothesis' [70]. When the tRNA concentration of minor codons becomes extremely limited, ribosomes of the host cell block at the minor codons to inhibite the ribosome from entering into the initiation site effectively, thereby resulting in a decrease in the translation rate. Moreover, the non-preferential codons locating at the translation initiation region modulate the number of ribosomes that are sequestered by an mRNA if the rates of elongation at these codons were so sufficiently slow that stalled ribosomes could block access to the initiation signals [19,71].

In summary, the conserved non-preferential codons in the translation initiation region have a high relationship with the regulation of gene expression. And the conserved codons with negative CUB are preferentially used in the initial region, which may be explained by the minor codon modulator hypothesis and the translation selection. These codons within this critical region might play a negative role in regulation of gene expression.

## List of abbreviations

PRRSV: Porcine reproductive and respitatory syndrome virus; SCUV: synonymous codon usage values; CUB: codon usage bias; US: Northern American isolate; EU: European isolate.

## Competing interests

The authors declare that they have no competing interests.

## Authors' contributions

JHS and XXM carried out the molecular genetic studies, participated in the sequence alignment and drafted the manuscript. YLH, JDL and XSM participated in the sequence alignment. YXD and XNL participated in the design of the study and performed the statistical analysis. XPC conceived of the study, and participated in its design and coordination and helped to draft the manuscript. All authors read and approved the final manuscript.
